# Contextual factors influencing the integration of physical activity policy, systems, and environmental interventions in the cooperative extension system: a systematic review

**DOI:** 10.1186/s12966-026-01927-8

**Published:** 2026-04-30

**Authors:** Shelly Palmer, Emily Shaw, Michelle Grocke-Dewey, Kelsay Corlew, Laura Flournoy, Laura E. Balis

**Affiliations:** 1 Center for Nutrition & Health Impact, Omaha, NE USA; 2 Department of Human Development and Community Health, Montana State University, Bozeman, MT USA

**Keywords:** Contextual inquiry, Implementation determinants, Physical activity, Policy, systems, and environmental interventions, Implementation outcomes, Implementation strategies, Community settings, Cooperative Extension

## Abstract

**Background:**

Physical activity policy, systems, and environmental changes (PSEs) are effective but complex public health interventions. PSEs are increasingly being implemented through Cooperative Extension, a national system historically focused on individual-level direct education. Researchers have begun conducting contextual inquiry studies, but the extent to which they have matched barriers and facilitators to specific implementation strategies is unknown. This study seeks to understand common barriers and facilitators to integrating physical activity PSEs in Extension and identify implementation strategies selected in response.

**Methods:**

A systematic review was conducted in June 2025 using three databases and two Extension-specific journals, with search terms focused on Extension; barriers, facilitators, and contextual factors; PSEs; and physical activity. Studies were included if they (1) were original, peer-reviewed articles, theses, or dissertations, (2) took place within Extension, (3) investigated integration of physical activity PSEs, (4) included barriers, facilitators, or contextual factors, (5) were in English, and (6) were published from 2014 to present. Two authors independently reviewed titles and abstracts for inclusion/exclusion, reviewed full text of remaining articles, and extracted data based on the Implementation Research Logic Model. Frequencies and proportions were calculated to tabulate data.

**Results:**

Thirteen studies met inclusion criteria. Outcomes of interest were most commonly implementation (*n* = 9, 69%), adoption (*n* = 3, 23%), and maintenance (*n* = 3, 23%). Contextual inquiry was most commonly based on the Consolidated Framework for Implementation Research (*n* = 5, 38%) or no framework (*n* = 5, 38%). Studies using a framework predominantly collected data on the individuals (*n* = 6, 46%) or outer setting (*n* = 6, 46%) domains. Common barriers were individual capability, local conditions, and intervention complexity (each *n* = 6, 46%). Common facilitators were partnerships and connections (*n* = 7, 54%) and individual capability (*n* = 6, 46%). Most studies (*n* = 8, 62%) provided recommendations based on findings, although these were not explicitly labeled as implementation strategies.

**Conclusions:**

Results provide implications for improving contextual inquiry and implementation strategy selection in Extension. Contextual inquiry should be based on a determinant framework to understand multi-level influences on PSE integration. Otherwise, critical barriers within the inner setting may be overlooked. Specific matching processes should be used to engage key individuals in selecting implementation strategies that address specific barriers.

**Supplementary Information:**

The online version contains supplementary material available at 10.1186/s12966-026-01927-8.

## Background

Physical activity policy, systems, and environmental changes (PSEs) are effective but complex public health interventions. PSEs are increasingly being implemented through Cooperative Extension (herein: Extension), a United States system historically focused on serving communities through practical, needs-based educational programs. The Extension system delivers programs through a three-tiered system providing support through a combination of federal, state, and county funding [1]. At the federal level, the United States Department of Agriculture (USDA) National Institute of Food and Agriculture distributes funding. At the state level, land-grant universities house Extension systems. At the local level, regional and county offices house Extension Agents or Educators (herein: Agents) to serve the public [2].

While structures differ across states, in general, Agents collaborate with land-grant university faculty and community members to offer educational workshops, programs, and consultations [2]. Many of the programs Extension provides are at the individual level (e.g., 4-H, pesticide applicator trainings, ServSafe, Dining with Diabetes, and Strong People) [2–5]. As National policies prioritize the need for PSEs to address community-level health [6–8], Extension professionals started adopting these interventions, including posting point-of-decision prompts [9], expanding bicycle infrastructure [10], and establishing out-of-school time physical activity guidelines [11, 12].

PSEs are more complex to implement than individual-level education interventions, as they require relationship and trust building with community groups to identify needs, can take many years to implement, and may not fit typical Extension evaluation processes [9, 12]. Over the last few years, researchers have begun conducting contextual inquiry to understand barriers and facilitators to integrating physical activity PSEs in Extension [13–15] and testing relevant implementation strategies [16]. In preparation for scaling out effective implementation strategies to additional state systems, work is needed to understand context across states. Implementation context can vary by state, as Extension systems operate autonomously, have different structures, cultures, and staffing models, and serve diverse communities across the country [17].

Implementation scientists have called for rapid methods to understand context in new or different settings to reduce the time and expense of conducing new contextual inquiry studies [18]. One recommended method is conducting systematic reviews to synthesize implementation determinants across settings [18]. Instead of conducting lengthy contextual inquiry studies, researchers could then conduct brief studies or use pragmatic process to assess which barriers and facilitators are relevant in additional settings.

A systematic review could also be used to aggregate implementation strategies used across settings to speed the implementation strategy selection process. These implementation strategies could serve as a starting point for recommended approaches to selecting and tailoring strategies to overcome barriers and capitalize on facilitators in each setting [16]. For example, a study may find that cost is a common barrier across settings and “leverage funding sources” (e.g., through mini-grants) [19] is a commonly used successful implementation strategy.

However, the extent to which scholars have matched barriers and facilitators to specific implementation strategies is unknown. Identifying relevant implementation strategies can be challenging, considering the limited literature on pragmatic selection processes, lack of training on identification and adaptation, and uncertainty around how many strategies are needed to address each barrier [20–22]. Thus, the primary goals of this systematic review were to identify (1) common barriers and facilitators to integrating PSEs in state Extension systems, (2) potential implementation strategies selected in response, and (3) how these implementation strategies were selected. A secondary goal of this review was to determine whether a systematic review of implementation determinants and strategies was a useful approach to conducting rapid contextual inquiry and speeding the implementation strategy selection process.

## Methods

### Data sources

Preferred Reporting Items for Systematic reviews and MetaAnalyses (PRISMA) guidelines were followed (see Additional File 1) [23]. The systematic review was conducted through searching PubMed, Academic Search Complete, and Open Access Thesis and Dissertation in June 2025 for terms focused on Extension; barriers, facilitators, or contextual factors; policy, systems and environmental changes; and physical activity. In addition, two non-PubMed indexed journals specific to Extension, the Journal of Human Sciences and Extension and Journal of Extension, were searched by hand. The research team considered including grey literature (e.g., impact statements, state Extension websites), as done previously [24]. Based on a preliminary review of these sources, the team determined that they were focused on program evaluation results and did not contain information on contextual inquiry. See Additional File 2 for the complete search strategy.

### Study selection

Studies were included if they (1) were original, peer-reviewed articles, theses, or dissertations (not conference abstracts), (2) took place within the United States Cooperative Extension System, (3) investigated the integration of physical activity policy, systems, or environmental interventions, (4) included barriers, facilitators, or contextual factors to integration, (5) were written in English, and (6) were published between 2014 and 2025 to align with inclusion of physical activity in the Farm Bill that funds Extension [25] and subsequent Extension National Framework for Health and Wellness [26]. Studies were excluded if they (1) were not original, peer-reviewed articles, theses, or dissertations, (2) did not take place within Unites States Cooperative Extension System, (3) did not investigate the integration of physical activity policy, systems, or environmental interventions, (4) did not include barriers, facilitators, or contextual factors to integration, (5) were not written in English, and (6) were published prior to 2014.

Two authors (ES and KC or ES and LF) independently reviewed each article’s title and abstract for inclusion or exclusion. Discrepancies were resolved through discussion among authors in team meetings. Next, for the included articles, two authors (ES and KC or ES and LF) independently reviewed the full text for inclusion or exclusion and noted reasons for exclusion. Discrepancies were discussed and resolved by authors in team meetings.

### Data extraction

Data extraction focused on key study characteristics and results based on the Implementation Research Logic Model (IRLM), a tool for planning and reporting implementation studies [27]. The IRLM is similar to typical program evaluation logic models, but is focused on documenting the linkages between core elements of implementation research: determinants, implementation strategies, mechanisms, and outcomes [27]. The theory underlying this causal modeling is that *determinants* (i.e., context-specific barriers and facilitators) of integrating an *intervention* inform the selection and tailoring of *implementation strategies*, which work through specific *mechanisms of action* to lead to *implementation outcomes* and, ultimately, clinical or community outcomes [27].

Within the *determinants* element, data was extracted on the theory, model, or framework used to conduct contextual inquiry and the levels or domains included, the type of study (qualitative, quantitative, mixed methods), who data was collected from (e.g., community members, Agents, administrators, partners), and the barriers or facilitators identified. Within the *intervention* element, data was extracted on the physical activity PSE intervention(s) of interest (general or specific). Data was also extracted on potential *implementation strategies*, how they were selected, and their *mechanisms* of action. Finally, data were extracted on antecedent (acceptability, appropriateness, feasibility) and primary *implementation outcomes* (reach, adoption, implementation cost and fidelity, maintenance, and scaling) [28–31]. See Additional File 3 for the data extraction guide.

To establish consistent coding and refine the coding guide, four authors (ES, KC, ES, LF) coded the first four articles. Authors met to resolve discrepancies and refine the coding guide. Next, two authors (SP and KC or ES and LF) independently extracted data from each article. Pairs of coders met to resolve discrepancies. Remaining discrepancies were resolved by authors at team meetings.

### Quality appraisal

The Mixed Methods Appraisal Tool (MMAT) version 2018 [32] was used to appraise article quality. The MMAT was selected for this review as it is validated to appraise different study designs, including qualitative, quantitative randomized, quantitative non-randomized, quantitative descriptive, and mixed methods. To begin, there are two screening questions: “Are there clear research questions?” and “Do the collected data allow to address the research questions?”. Next, based on the study design there are five questions asking about the quality of the methods to answer the research questions. For each question there are response options of “yes”, “no”, and “can’t tell”. Two researchers (KC and LF) used the MMAT to independently assess each article. Areas of disagreement were noted and resolved by authors at team meetings. The number of “yes” responses were calculated to provide a quality score for each article.

### Data synthesis

All included studies were synthesized together. To prepare the data for synthesis, implementation determinants as reported by study authors were coded with the most relevant construct from the Consolidated Framework for Implementation Research (CFIR) [33]. CFIR is a commonly used determinant framework that includes constructs in five domains: (1) inner setting, (2) outer setting, (3) individuals, (4) implementation process, and (5) innovation [33]. Next, implementation strategies (including suggestions for improving PSEs integration that were not specifically labeled as implementation strategies) as reported by study authors were coded with the most relevant strategy from the Implementation Strategies Applied in Communities (ISAC) compilation [19]. Coding was completed independently by pairs of authors, reconciled, and reviewed by the first author. Finally, frequencies and proportions of all variables were calculated to tabulate the data.

## Results

### Selection of sources

**Fig. 1:**
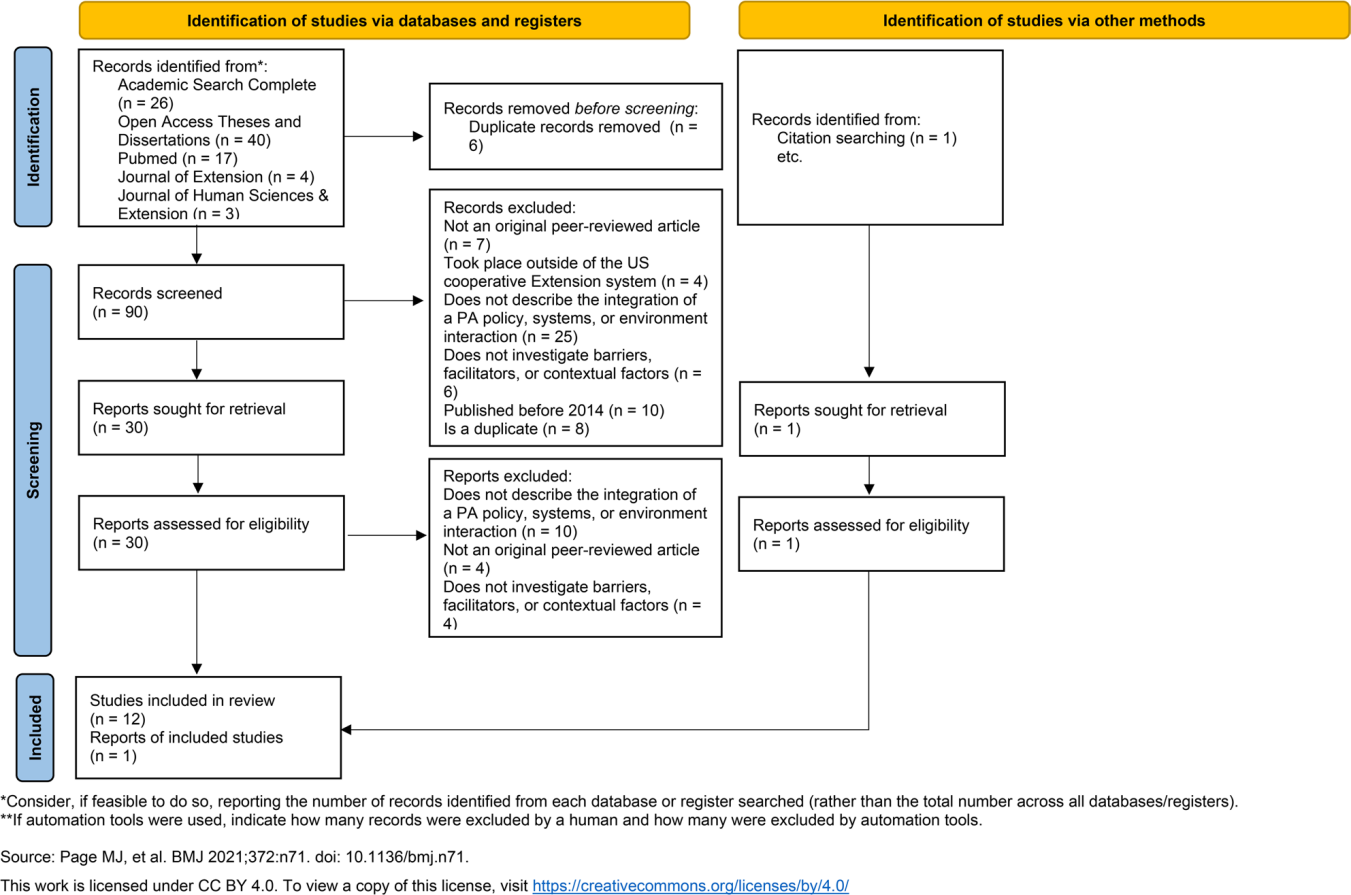
PRISMA flow diagram

The initial search for peer-reviewed articles yielded 90 articles after six duplicate articles were removed. After title and abstract review, 60 were excluded because they (1) they did not describe the integration of a physical activity policy, systems, or environmental interventions (*n* = 25), (2) were published before 2014 (*n* = 10), (3) were a duplicate (*n* = 8), (4) were not an original peer reviewed article, thesis, or dissertation (*n* = 7), (5) did not investigate barriers, facilitators, or contextual factors (*n* = 6), and (6) took place outside of Extension (*n* = 4). After full text review of the remaining 30 articles, 17 were excluded because they did not describe the integration of a physical activity policy, system, or environment interaction (*n* = 10), were not an original peer reviewed article, thesis, or dissertation (*n* = 4), and did not investigate barriers, facilitators, or contextual factors (*n* = 4). A total of 13 peer reviewed articles, theses, or dissertations (herein: articles) met inclusion criteria for this review. See the PRISMA flow diagram (Fig. 1). 

### Characteristics of sources

Table 1 reports the characteristics of the studies, and full extracted information in Additional File 4. The studies were conducted across all Extension Regions [34], with the majority (*n* = 8, 62%) from the Southern region [13, 15, 35–40]. Some studies took place across more than one region. Most of the studies were conducted in rural settings (*n* = 9, 69%) [14, 15, 35, 37–42]. The program funding sources included general Extension funding (*n* = 7, 54%) [13, 14, 37, 40–43], multiple funding sources (*n* = 3, 23%) [15, 36, 39], High Obesity Program (HOP) (*n* = 2, 15%) [35, 38], and the Supplemental Nutrition Assistance Program-Education (SNAP-Ed) (*n* = 1, 8%) [44].

**Table 1:** Study characteristics of original research included in a systematic review of barriers and facilitators in Extension systems implementing policy, systems, and environment interventions (*n* = 13)

Characteristics	*n* (%)
State^a^	
Southern	8 (62)
Western	4 (31)
Northeast	2 (15)
North Central	1 (8)
Rurality	
Rural	9 (69)
Rural and urban	2 (15)
Not mentioned	2 (15)
Study type	
Qualitative	8 (62)
Quantitative	3 (23)
Mixed methods	2 (15)
Intervention type	
Physical activity and nutrition PSEs	11 (85)
Physical activity PSEs only	2 (15)
Extension program funding	
General	7 (54)
Multiple funding sources	3 (23)
HOP	2 (15)
SNAP-Ed	1 (8)
Study participants	
Implementers (e.g., Extension Agents)	9 (69)
Community partners (e.g., coalition members)	2 (15)
Multiple participants	2 (15)
Implementation outcomes of interest^a^	
Implementation	9 (69)
Maintenance	3 (23)
Adoption	3 (23)
Acceptability	2 (15)
Feasibility	2 (15)
None reported	2 (15)
Cost	1 (8)
Fidelity	1 (8)
Reach	1 (8)
Determinant framework	
CFIR	5 (38)
No framework	5 (38)
Other (e.g., process model or behavior change theory)	3 (23)
Data collection domains^a^	
Individual	6 (46)
Outer	6 (46)
Innovation	4 (31)
Inner	4 (31)
Process	1 (8)
Not specified	2 (15)
Not applicable (no framework)	5 (38)
Implementation strategy selection process^a^	
Provided recommendations based on results	8 (62)
Specified implementation strategies	3 (23)
Did not provide recommendations or specify implementation strategies	2 (15)
Implementation strategies selected^a^	
Provide training	5 (38)
Conduct pragmatic evaluation	3 (23)
Provide resources	3 (23)
Provide technical assistance	2 (15)
Conduct local needs assessment	2 (15)
Facilitate peer learning	2 (15)
Develop a coalition or workgroup	2 (15)
Leverage funding sources	2 (15)
Enhance staffing	2 (15)
Assess for readiness and identify barriers and facilitators	1 (8)
Conduct local consensus discussions	1 (8)
Increase demand	1 (8)
Create program guide	1 (8)
Engage community members	1 (8)
Tailor recruitment strategies	1 (8)
Choose strategic partner organizations	1 (8)
Engage potential partners	1 (8)
Incentive delivery agents	1 (8)
Change programming focus	1 (8)
Conduct demonstration events	1 (8)
Change delivery agent roles	1 (8)

^a^ Percentages may be more than 100, as multiple characteristics could be reported

### Study designs and data collection

The majority of the studies were qualitative (*n* = 8, 62%) [13, 15, 35, 38, 39, 41, 42, 44], with fewer quantitative (*n* = 3, 15%) [36, 40, 43] or mixed methods designs (*n* = 2, 15%) [14, 37] (Table 1). Most investigated integration of physical activity and nutrition PSEs collectively (*n* = 11, 85%) [15, 35–44], with fewer focused specifically on physical activity PSEs (*n* = 2, 15%). Most studies collected and reported data from program implementers (e.g., Agents) (*n* = 9, 69%) [13–15, 35, 37, 40, 41, 43, 44], with fewer reporting data from community partners (*n* = 2, 15%) [38, 42] or multiple participants (e.g., implementers, community partners, and community members) (*n* = 2, 15%) [36, 39]. The implementation outcomes of interest were most commonly implementation (*n* = 9, 69%) [13–15, 35, 37, 39–41, 44], followed by adoption (*n* = 3, 23%) [13, 36, 37], and maintenance (*n* = 3, 23%) [13, 15, 39]. Contextual inquiry was most commonly based on the CFIR (*n* = 5, 38%) [13–15, 37, 40], not based on a framework (*n* = 5, 38%) [35, 36, 41, 42, 44], or based on a theory other than a determinant framework (*n* = 3, 23%) [38, 39, 43]. Studies that used a framework most commonly collected data on individuals (*n* = 6, 46%) [13–15, 40, 42, 43] or outer setting (*n* = 6, 46%) domains [13–15, 37, 40, 42], followed by the innovation (*n* = 4, 31%) [13–15, 40] and inner setting (*n* = 4, 31%) [14, 15, 40, 42] domains.

Related to the quality appraisal of the articles, studies scored on average 4 and ranged from 0 to 7 (possible range of 0–7, with 0 indicating the lowest score and 7 indicating the highest score). Quality review decisions are in Additional File 4.

### Reported barriers and facilitators

The reported barriers and facilitators within each CFIR domain are reported in Table 2. Nine studies (69%) reported results for the individuals domain. Within this domain, barriers were most commonly related to innovation deliverers’ capability (e.g., low confidence, knowledge, or sense of direction for conducting PSE work) (*n* = 6, 46%) [13, 15, 35, 40, 43, 44], motivation (*n* = 3, 23%) [13, 15, 40], and opportunity (*n* = 3, 23%) [13, 35, 37]. Facilitators included innovation deliverers’ capability (e.g., high levels of knowledge, leadership skills, confidence, or comfort with PSE work) (*n* = 6, 46%) [13, 39, 40, 42–44] and motivation (*n* = 4, 31%) [13, 15, 36, 40].

**Table 2:** The number of studies reporting domains and constructs within the Consolidated Framework for Implementation Research (CFIR) that influence the integration of policy, systems, and environmental changes in Extension systems (*n* = 13)

CFIR Domains and Constructs^a^	Barriers *n* (%)	Facilitators *n* (%)
**Individuals**	**9 (69)**	**9 (69)**
Innovation deliverers: capability	6 (46)	6 (46)
Innovation deliverers: motivation	3 (23)	4 (31)
Innovation deliverers: opportunity	3 (23)	1 (8)
Innovation recipients: motivation	1 (8)	1 (8)
Implementation team members: motivation	1 (8)	1 (8)
Innovation recipients: need	0 (0)	2 (15)
Implementation team members: opportunity	1 (8)	0 (0)
Innovation deliverers: need	0 (0)	1 (8)
Mid-level leaders: capability	0 (0)	1 (8)
Other implementation support: motivation	0 (0)	1 (8)
Other implementation support: capability	0 (0)	1 (8)
**Outer setting**	**8 (62)**	**9 (69)**
Partnerships & connections	5 (38)	7 (54)
Local conditions	6 (46)	3 (23)
Local attitudes	4 (31)	4 (31)
Financing	3 (23)	2 (15)
Policies & laws	2 (15)	1 (8)
Critical incidents	1 (8)	1 (8)
**Inner setting**	**7 (54)**	**9 (69)**
Access to knowledge & information	1 (8)	4 (31)
Funding	3 (23)	1 (8)
Mission alignment	2 (15)	2 (15)
Communications	1 (8)	3 (23)
Relative priority	2 (15)	1 (8)
Available resources	2 (15)	1 (8)
Culture	0 (0)	3 (23)
Relational connections	0 (0)	3 (23)
Work infrastructure	3 (23)	0 (0)
Incentive systems	2 (15)	0 (0)
Deliverer centeredness	1 (8)	1 (8)
Structural characteristics	1 (8)	0 (0)
Learning centeredness	1 (8)	0 (0)
Information technology infrastructure	1 (8)	0 (0)
Implementation climate	0 (0)	1 (8)
Leadership engagement	0 (0)	1 (8)
Compatibility	0 (0)	1 (8)
**Innovation**	**8 (62)**	**5 (38)**
Complexity	6 (46)	1 (8)
Design	1 (8)	1 (8)
Relative advantage	1 (8)	1 (8)
Evidence base	0 (0)	2 (15)
Cost	1 (8)	0 (0)
Adaptability	0 (0)	1 (8)
**Process**	**1 (8)**	**2 (15)**
Engaging	1 (8)	0 (0)
Reflecting and evaluating	1 (8)	0 (0)
Doing	1 (8)	0 (0)
Adapting	0 (0)	1 (8)
Teaming	0 (0)	1 (8)

^a^ Percentages may be more than 100, as multiple domains and constructs could be reported

Nine studies (69%) [13–15, 35–37, 39, 42, 43] reported results for the outer setting domain. Common barriers included local conditions (*n* = 6, 46%) (e.g., existing physical activity environment, engagement of community members, limited financial resources, inclement weather) [13, 15, 38, 41, 42, 44], partnerships and connections (*n* = 5, 38%) [13, 15, 35, 38, 44], local attitudes (*n* = 4, 31%) [13, 15, 38, 42], and financing (*n* = 3, 23%) [13, 15, 37]. Facilitators primarily included partnerships and connections (e.g., having partners who were ready for change, developing partnerships with community partners who had existing resources) (*n* = 7, 54%) [13–15, 35–37, 43], local attitudes (*n* = 4, 31%) [15, 16, 35, 39], and local conditions (*n* = 3, 23%) [13, 15, 43].

Nine studies (69%) [14, 15, 35–40, 43] reported results for the inner setting domain. Common barriers included funding (e.g., identifying specific sources of funding for PSE-related work) (*n* = 3, 23%) [15, 37, 42] and work infrastructure (e.g., staff turnover, limited capacity, and conflicting responsibilities) (*n* = 3, 23%) [15, 39, 44]. Facilitators primarily included access to knowledge and information (e.g., presences of experienced staff and adequate training) (*n* = 4, 31%) [15, 35, 37, 38], communications (*n* = 3, 23%) [15, 38, 39], culture (*n* = 3, 23%) [39, 40, 43], and relational connections (*n* = 3, 23%) [35, 38, 39].

Eight studies (64%) [13–15, 35, 39–41, 44] reported results for the innovation domain. The most frequently reported barrier was complexity (e.g., PSE work is long-term and can take many years, requires more planning and time, relies on partnerships, requires specialized knowledge, and is difficult to report outcomes on) (*n* = 6, 46%) [13, 15, 35, 40, 41, 44]. The most frequently reported facilitator was evidence base (e.g., PSE work has a positive impact on the health of the community and has the potential to reach many community members) (*n* = 2, 15%) [36, 40].

Two studies (15%) [41, 42] reported results for the process domain. One [42] reported barriers of engaging (e.g., contacting community partners) (*n* = 1 8%), reflecting and evaluating (*n* = 1, 8%), and doing (*n* = 1, 8%). Facilitators included adapting (e.g., tailoring the PSE activities to fit the community) (*n* = 1, 8%) [41] and teaming (e.g., facilitating strong, diverse groups of partners) (*n* = 1, 8%) [42].

### Implementation strategies identified

Two studies (15%) did not specify implementation strategies or provide recommendations for improving physical activity PSE integration. Eight studies (62%) [35–37, 39–42, 44] provided recommendations for improving the integration of PSEs, although they did not specifically mention implementation strategies (Table 3). Three studies (23%) [13–15] used a specific process to select and report potential implementation strategies. The first of these studies identified barriers related to individuals’ motivation, capability, and opportunity; innovation complexity; and multiple outer setting factors [13]. Study results, existing literature, and an Integrated-Research Practice Partnership approach were used to select implementation strategies (*provide resources*,* provide technical assistance*,* leverage funding sources*) in collaboration with Extension Agents [13]. The second study identified cost, relative priority, and available resources as barriers, and used the CFIR-Expert Recommendations for Implementing Change (ERIC) matching tool to match these constructs with multiple implementation strategies from the ERIC compilation: conduct local needs assessment, facilitate peer learning, develop a coalition or workgroup, assess for readiness and identify barriers and facilitators, conduct local consensus discussions, and increase demand [14]. The third study found individuals’ capability and motivation, community members’ motivation, innovation complexity, and several inner and outer setting factors as barriers [15]. The authors provided potential implementation strategies (*provide resources*,* leverage funding sources*,* provide training*,* incentivize delivery agents*) based on study results and described a subsequent study to be conducted to prioritize them [15]. See Table 3 for more details. Overall, the most frequently recommended implementation strategies across all studies were *provide training* (*n* = 5, 38%) [15, 35, 37, 40, 44], *provide resources* (*n* = 3, 23%) [13, 15, 41], and *conduct pragmatic evaluation* (*n* = 3, 23%) [37, 40, 41].

**Table 3:** Implementation outcomes, contextual factors, and implementation strategies reported in original research included in a systematic review of integration of policy, systems, and environmental changes in state Extension systems

First Author, Year	Study objective	Implementation outcomes of interest	Barriers	Facilitators	Implementation strategy selection	Implementation strategies identified
Intervention type						
Balis, 2023 Physical activity PSE [13]	Inform implementation strategies through understanding Agents’ perceptions of (1) built environment approaches, (2) a toolkit developed as part of the CDC’s High Obesity Program, and 3) support needed to implement built environment approaches.	Adoption, implementation, maintenance, cost, fidelity	*Individuals*:^1^ Motivation, Capability, Opportunity *Innovation*: Complexity *Outer setting*: Local attitudes, Local conditions, Partnerships & connections, Financing	*Individuals*: Capability, *Innovation recipients*: need, Motivation *Outer setting*: Financing, Partnerships & connections, Local conditions	Specified implementation strategies selected through study results, existing literature, and Integrated Research-Practice Partnership recommendations	Provide resources, Provide technical assistance, Leverage funding sources
Balis, 2022 Physical activity PSE [14]	Understand (1) barriers and facilitators to implementing built environment approaches in two state Extension systems, (2) preferences for potential implementation strategies, and (3) preferences for micro-level built environment approaches.	Implementation	*Innovation*: Cost *Inner setting*: Relative priority, Available resources	*Inner setting*: Implementation climate *Outer setting*: Policies & laws, Local attitudes	Specified implementation strategies selected through CFIR-ERIC matching tool	Conduct local needs assessment, Facilitate peer learning, Develop a coalition or workgroup, Assess for readiness and identify barriers and facilitators^2^, Conduct local consensus discussions^2^, Increase demand^2^
Bressler, 2019 Physical activity PSE	Determine the best strategies to prepare Family Consumer Science (FCS) Extension Agents to conduct PSE work to reduce the prevalence of obesity in other rural communities.	Implementation	*Individual*: Opportunity, Capability *Innovation*: Complexity *Outer setting*: Partnerships & connections, Policies & laws	*Inner setting*: Access to knowledge & information, Relational connections *Outer setting*: Partnerships & connections, Local attitudes	Provided recommendations based on study results	Provide training, Engage potential partners, Enhance staffing
Brooks, 2024 Nutrition and physical activity PSE [36]	Explore factors influencing the adoption of direct education programs and PSE change initiatives focused on physical activity for SNAP-eligible audiences by Virginia EFNEP and SNAP-Ed staff.	Adoption	None identified	*Individuals*: Motivation *Innovation*: Evidence base *Inner setting*: Compatibility, Mission alignment *Outer setting*: Partnerships & connections	Provided recommendations based on study results and existing literature	Change programming focus
Houghtaling, 2023 Nutrition and physical activity PSE [15]	Identify Louisiana Cooperative Extension Service FCS practitioners’ barriers and facilitators to the implementation of healthy eating and active living PSE changes in rural Louisiana communities.	Implementation, maintenance	*Individuals*: Capability, Motivation, *Implementation team members* (community members): motivation *Innovation*: Complexity *Inner setting*: Communications, Access to knowledge & information, Deliverer-Centeredness, Learning-Centeredness, Available resources, Funding, Work infrastructure, Information technology infrastructure, Incentive systems *Outer setting*: Partnerships & connections, Local conditions, Financing, Local attitudes, Critical incidents, Policies & laws	*Individuals*: Motivation, *Implementation team members*: motivation *Innovation*: Complexity, Adaptability *Inner setting*: Communications, Access to knowledge & information, Deliverer-Centeredness, Available resources, Funding *Outer setting*: Partnerships & connections, Local conditions, Financing, Local attitudes, Critical incidents	Described additional research being conducted to select implementation strategies through prioritization. Provided recommendations based on study results.	Provide resources, Leverage funding sources, Provide training, Incentivize delivery agents
Kennedy, 2025 Nutrition and physical activity PSE [37]	Understand FCS Extension agents’ perceptions of PSE change supports, benefits for their communities, and resources and partnerships important for implementation.	Adoption, implementation	*Individuals*: Opportunity *Inner setting*: Structural characteristics, Funding *Outer setting*: Financing	*Innovation*: Relative advantage *Inner setting*: Leadership engagement, Access to knowledge & information *Outer setting*: Partnerships & connections	Provided recommendations based on study results.	Conduct demonstration events, Provide training, Conduct pragmatic evaluation
Losavio, 2022 Nutrition and physical activity PSE [38]	Investigate motivations among sustained members of coalitions facilitated by LSU AgCenter Extension agents in High Obesity Program target parishes.	None	*Outer setting*: Local conditions, Local attitudes, Partnerships & connections	*Individuals*: *Other implementation support* (coalition members): motivation, *Other implementation support* (coalition members): capability *Inner setting* (coalitions): Communication, Access to knowledge & information, Relational connections	Not reported	Not reported
Lu, 2017 Nutrition and physical activity PSE [43]	Apply the Theory of Planned Behavior to examine the relationship between the constructs of background factors and beliefs towards using PSE strategies and reported use of PSE strategies to prevent obesity by a group of professional nutrition educators.	None	*Individuals*: Capability	*Individuals*: Capability, Opportunity, *Mid-level leaders*: capability *Inner setting*: Culture *Outer setting*: Local conditions, Partnerships & connections	Not reported	Not reported
Meier, 2023 Nutrition and physical activity PSE [44]	Explore the use of community engagement as a strategy to address the common challenges experienced in implementing a multi-component community-based program.	Implementation	*Individuals*: Capability *Innovation*: Complexity *Inner setting*: Work infrastructure *Outer setting*: Partnerships & connections, Local conditions	*Individuals*: Capability, Need	Provided recommendations based on study results.	Conduct local needs assessment, Enhance staffing, Provide training
Pardo, 2018 Nutrition and physical activity PSE [39]	Provide information about various processes and factors related to an academic-faith based organization partnership established for the purposes of implementing a faith-based nutrition/physical activity education program and establishing healthy policy and environmental changes at the church.	Implementation, maintenance, acceptability	*Individuals*: *Innovation recipients*: motivation *Innovation*: Design *Inner setting*: Work infrastructure, Mission alignment	*Individuals*: Capability, *Innovation recipients*: need *Outer setting*: Local attitudes *Inner setting*: Relational connections, Communication, Culture	Provided recommendations based on study results and researcher experience	Engage community members, tailor recruitment strategies, develop a coalition or workgroup
Seguin-Fowler, 2020 Physical activity PSE [41]	Describe the implementation and feasibility of eHEART (Healthy Eating and Activity in Rural Towns), a civic engagement curriculum adapted for online dissemination.	Implementation, feasibility	*Innovation*: Complexity *Outer setting*: Local conditions *Process*: Reflecting & evaluating, Engaging, Doing	*Individuals*: *Innovation recipients*: motivation *Innovation*: Design *Process*: Adapting	Provided recommendations based on study results.	Provide resources, Conduct pragmatic evaluation, Create program guide
Seguin, 2018 Nutrition and physical activity PSE [42]	Assess the feasibility and effectiveness of a civic engagement curriculum designed to engage rural residents in improving their local food or physical activity environment.	Feasibility	*Individuals*: *Implementation team members* (change club members): opportunity *Inner setting* (change clubs): Funding *Outer setting*: Local attitudes, Local conditions	*Individuals*: Capability *Outer setting*: Partnerships & connections *Process*: Teaming	Provided recommendations based on study results.	Choose strategic partner organizations, Facilitate peer learning
Washburn, 2022 Nutrition and physical activity PSE [40]	Explore perceived acceptability of PSE change work among FCS Extension agents in two states and broadly examine potential barriers and facilitators to advancing Extension’s PSE change work.	Implementation, acceptability	*Individuals*: Capability, Motivation *Innovation*: Complexity, Relative advantage *Inner setting*: Relative priority, Incentive systems, Mission alignment	*Individuals*: Capability, Motivation *Innovation*: Evidence base *Inner setting*: Mission alignment, Relative priority, Culture	Provided recommendations based on study results.	Conduct pragmatic evaluation, Provide training, Provide technical assistance, Change delivery agent roles

^1^Unless otherwise specified, individual characteristics refer to Innovation deliverers. Other project roles (e.g., Implementation team members) are underlined prior to the relevant characteristic

^2^Implementation strategies from the Expert Recommendations for Implementing Change (ERIC) compilation. All other implementation strategies are from the Implementation Strategies Applied in Communities (ISAC) compilation

## Discussion

The goal of this study was to understand common barriers and facilitators to integrating physical activity PSEs in Extension and identify implementation strategies selected in response. All the studies included in the review reported at least one facilitator and/or barrier, most commonly from the individuals domain of the CFIR. The majority of the articles provided implementation strategy recommendations based on study results, although they did not label them as implementation strategies. Thus, while data to answer the research questions were present, results should be interpreted with caution due to the methods used, which may not have led to robust contextual inquiry and data-driven implementation strategy selection. The findings lead to implications for improving contextual inquiry and implementation strategy selection in Extension, including using determinant frameworks to understand multi-level influences on PSE integration and aligning implementation outcomes and strategies with standard implementation science terminology.

First, contextual inquiry studies could generate better data through applying an implementation science determinant framework. Most of the studies in this review were rated as moderate quality; a common issue was that the methods were often not appropriate to answer the research question. This was often because the researchers either did not use a framework or applied theories, models, and frameworks other than determinant frameworks, such as the Social Cognitive Theory [38, 45] or Theory of Planned Behavior [43, 46]. These theories are designed to understand how individuals make decisions related to their own health behaviors and are insufficient for understanding individuals’ behavior within the context of organizations they work in [45, 46]. In addition, determinant frameworks assess multi-level contextual factors influencing implementation beyond the individual level, with most including determinants related to the evidence-based intervention, organization it is implemented in, and external environment [47].

In this review, studies most often reported barriers and facilitators at the individuals level. These data revealed that capability and motivation of Agents (innovation deliverers) is viewed as both a barrier and a facilitator. However, this may be overreported due to the use of individual-level health behavior frameworks or no framework. Using multi-level determinant frameworks to structure contextual inquiry studies may lead to a better understanding of barriers and facilitators beyond individual-level factors. For example, studies may reveal important factors in the inner setting domain, which is crucial for understanding how to integrate a new intervention – especially in organizations that are not focused solely on public health [19]. In addition, most studies included only the perceptions of Agents; none included perceptions of leaders or administrators, who may be more likely to contribute contextual details related to the inner setting [48].

Contextual inquiry studies lead to the selection of relevant implementation strategies to overcome barriers and capitalize on facilitators. The first step in the process is identifying the implementation outcomes of interest – for example, is the purpose of the study to understand barriers to and intervene to improve adoption, or maintenance? This is important as the contextual factors and implementation strategies differ by desired outcome [19, 49]. In the studies included in this review, the primary outcome of interest was implementation, with other adoption and maintenance not commonly reported. This is likely because many of the studies focused on personnel and systems already implementing PSEs. However, a greater focus on adoption may have been warranted, as there are many Extension personnel who are not yet working on physical activity PSEs although they could be.

Finally, the studies in the review suggested over 20 different implementation strategies. *Provide training* was the most commonly suggested; this is unsurprising given Extension’s historical focus on community education [1] and the prevalence of training and educational meetings as implementation strategies [50, 51]. However, there is limited evidence that training is an effective implementation strategy, unless it is ongoing or paired with other strategies (e.g., technical assistance, defined as the sharing of expertise for improving capacity to achieve outcomes [52]) [51, 53]. *Conduct pragmatic evaluation* was suggested in multiple articles, and is a recently identified strategy in the new ISAC compilation [19]. This is a promising strategy for Extension, as evaluating PSEs are more complex than evaluating direct education interventions [54–56]. Pragmatic evaluation methods, such as brief, valid data collection instruments, could provide Extension practitioners with feasible options that do not require research expertise, multiple days of observation, or costly data collection tools [19, 57].

However, it is difficult to determine if the implementation strategies in the included articles are likely to address barriers, capitalize on facilitators, and improve implementation outcomes, as most were not selected through a systematic process. Recommended implementation strategy selection processes typically include several steps, such as reviewing available information on the intervention and conducting a contextual assessment, identifying existing implementation strategies that are already in place, using matching tools to select new implementation strategies to overcome barriers and leverage facilitators, and tailoring the implementation strategies to the audience [16]. Relying on study results to identify implementation strategies without considering existing literature or completing additional participatory research may result in strategies that are not effective or are a poor fit for the setting.

A secondary goal of this study was to determine whether a systematic review of implementation determinants and strategies was a useful approach to conducting rapid contextual inquiry and speeding the implementation strategy selection process. Although there were noted limitations in the identification of contextual factors, and most barriers and facilitators were at the individuals level, the results could still be useful in understanding similar barriers and facilitators across states. The barriers and facilitators identified in a systematic review such as this could serve as a first step to facilitating a pragmatic process (e.g., a walking vote [16]) or developing a brief survey to identify common and unique barriers in new settings [18, 58].

A brief survey such as the Pragmatic Context Assessment Tool (pCAT) [59] could be used for this step. The pCAT assesses 10 CFIR constructs across four domains (inner setting, outer setting, process, and innovation characteristics) [59]. Interestingly, although the pCAT was developed based on common barriers and facilitators in clinical settings, the contextual factors identified in this review aligned with seven of the 10 pCAT constructs. Additional items could be added to the pCAT to reflect the most salient determinants in the review that were not included (e.g., local conditions, innovation complexity) and the survey could be adapted for community (instead of clinical) context and terminology [60]. Focusing on the four CFIR domains included in the pCAT could overcome the barrier of existing contextual inquiry studies focusing on individual-level determinants.

Lastly, the results of our systematic review may be less useful in identifying common implementation strategies (compared to implementation determinants) since they were not typically selected through specific processes or using existing compilations. There is a need to carefully select relevant implementation strategies and name them using standard compilations to build evidence across studies [19]. Taken together, conducting a systematic review based on the IRLM as a form of rapid contextual inquiry may be more useful in clinical settings that have a longer history of implementation science integration and investigations, as the included studies may be more likely to align with standard implementation science methods, frameworks, and taxonomies.

### Limitations

The strengths of this systematic review included use of the IRLM to structure data collection and use of the CFIR and ISAC compilation to synthesize data on implementation determinants and strategies, respectively. However, the varying terminology used across studies to refer to contextual factors, implementation outcomes, and implementation strategies is also a limitation. The research team coded contextual factors and implementation strategies according to consistent implementation science terms, but they may have been categorized by the original authors differently and meaning may have been lost. Moreover, we recognize there are other novel implementation strategies happening within Extension that were not captured in this review [61–63], as the articles they are reported in did not include contextual inquiry.

Finally, another limitation is that most studies included in this review investigated context related to nutrition and physical activity PSEs collectively. Implementing PSEs – whether focused on nutrition or physical activity – requires similar processes, including assessing needs and collaborating with community partners and decision-makers [62, 63]. Although the two physical activity-focused studies included in this review found similar barriers and facilitators to the other 11 studies, contextual factors specific to physical activity (e.g., community members’ physical activity preferences) may have been missed.

In addition, at the organizational level, nutrition PSEs interventions in general are considered a better “fit” for Extension work than physical activity, as they align with the system’s historical focus on agriculture [55]. Nutrition is also the primary focus of the SNAP-Ed program [64], which initiated Extension’s integration of both nutrition and physical activity PSEs [37]. Related, federal funding for SNAP-Ed ended in 2025 [65], which may lead to fewer PSEs implemented within Extension. Future work should explore who is implementing PSEs and to what extent within the new federal funding landscape.

## Conclusions

This systematic review aimed to understand common barriers and facilitators to integrating physical activity PSEs in Extension and identify implementation strategies selected in response. The review identified a total of 45 contextual factors and 21 implementation strategies across 13 studies. Results underscore the importance of improving contextual inquiry and implementation strategy selection within Extension. Few of the studies applied a determinant framework to identify barriers and facilitators, which is essential to understanding the multi-level influences on PSE integration. The individuals level was the most commonly reported construct, which leaves out critical barriers within the inner setting. Once contextual factors are identified, additional work is needed to apply formal implementation strategy matching processes that address specific barriers and leverage facilitators. In order to confirm barriers and facilitators in other settings, the next steps are to strategically select and measure contextual factors using pragmatic tools or processes.

## Supplementary Information

Additional file 1.

Additional file 2.

Additional file 3.

Additional file 4.

## Data Availability

All data generated or analyzed during this study are included in this published article and its supplementary information files. Raw data is included in Additional file 4.

## Abbreviations

CFIR: Consolidated Framework for Implementation Research
HOP: High Obesity Program
IRLM: Implementation Research Logic Model
ISAC: Implementation Strategies Applied in Communities
MMAT: Mixed Methods Appraisal Tool
pCAT: Pragmatic Context Assessment Tool
PRISMA: Preferred Reporting Items for Systematic reviews and MetaAnalyses
PSEs: Policy, Systems, and Environmental changes
SNAP-Ed: Supplemental Nutrition Assistance Program Education
USDA: United States Department of Agriculture
