# Supplementary material for: Contextual factors influencing the integration of physical activity policy, systems, and environmental interventions in the cooperative extension system: a systematic review
Source: Int J Behav Nutr Phys Act. 2026 Apr 30;23:63. doi: 10.1186/s12966-026-01927-8 (PMC13277215; doi:10.1186/s12966-026-01927-8)
Supplement: Supplementary file 2 — Additional file 2. [file 12966_2026_1927_MOESM2_ESM.pdf]

## Search Strategy

### PubMed

### Title Abstract Search

((("PSE change"[Title/Abstract] OR "policy, systems, environment"[Title/Abstract] OR "policy, systems, and environmental change"[Title/Abstract] OR "policy, systems, and environmental (PSE) change"[Title/Abstract] OR "PSE Work"[Title/Abstract] OR "Environmental Change Initiative*"[Title/Abstract] OR "built environment"[Title/Abstract] OR "PSE Initiatives"[Title/Abstract] OR PSE[Title/Abstract]) AND (facilitat*[Title/Abstract] OR barrier[Title/Abstract] OR motivator*[Title/Abstract] OR "Context* factor*"[Title/Abstract] OR "Implementation Strateg*"[Title/Abstract] OR "Implementation science"[Title/Abstract] OR "Consolidated Framework for Implementation Research"[Title/Abstract] OR CFIR[Title/Abstract] OR approach*[Title/Abstract] OR capacity[Title/Abstract] OR adopt[Title/Abstract] OR "change strateg*"[Title/Abstract] OR implementation[Title/Abstract])) AND ("Cooperative Extension"[Title/Abstract] OR "Cooperative Extension Service"[Title/Abstract] OR CES[Title/Abstract] OR Extension[Title/Abstract] OR "extension agent*"[Title/Abstract] OR "cooperative extension educat*"[Title/Abstract] OR "cooperative extension system"[Title/Abstract] OR "extension personnel"[Title/Abstract])) AND ("physical activit*"[Title/Abstract] OR "physical activity"[Title/Abstract] OR exercise*[Title/Abstract] OR exercising[Title/Abstract] OR workout*[Title/Abstract] OR "physical fitness"[Title/Abstract] OR "active living"[Title/Abstract] OR "health education"[Title/Abstract] OR "public health"[Title/Abstract])

### Academic Search Complete

### XB Title and Abstract

"PSE change" OR "policy, systems, environment" OR "policy, systems, and environmental change" OR "policy, systems, and environmental (PSE) change" OR "PSE Work" OR "Environmental Change Initiative*" OR "built environment" OR "PSE Initiatives" OR PSE

AND facilitat* OR barrier OR motivator* OR "Context* factor*" OR "Implementation Strateg*" OR "Implementation science" OR "Consolidated Framework for Implementation Research" OR CFIR OR approach* OR capacity OR adopt OR "change strateg*" OR implementation OR "motivation (Psychology)"

AND "Cooperative Extension" OR "Cooperative Extension Service" OR CES OR Extension OR "extension agent*" OR "cooperative extension educat*" OR "cooperative extension system" OR "extension personnel" OR "Expanded Food and Nutrition Education Program" OR ENFP OR "Supplemental Nutrition Assistance Program-Education"

AND "physical activit*" OR "physical activity" OR exercise* OR exercising OR workout* OR "physical fitness" OR "active living" OR "health education" OR "public health" OR "Physical Activity Direct Education"

**Open Access Thesis and Dissertation**

("EXTENSION AGENTS" OR "Cooperative Extension") AND ("physical activity" OR obesity)

**Journal of Human Sciences and Extension**

**Journal of Extension**

**Abstracts**

"PSE change" OR "policy, systems, environment" OR "policy, systems, and environmental change" OR "policy, systems, and environmental (PSE) change" OR "PSE Work" OR "Environmental Change Initiative*" OR "built environment" OR "PSE Initiatives" OR PSE

AND facilitat* OR barrier OR motivator* OR "Context* factor*" OR "Implementation Strateg*" OR "Implementation science" OR "Consolidated Framework for Implementation Research" OR CFIR OR approach* OR capacity OR adopt OR "change strateg*" OR implementation OR motivation
