# Supplementary material for: Contextual factors influencing the integration of physical activity policy, systems, and environmental interventions in the cooperative extension system: a systematic review
Source: Int J Behav Nutr Phys Act. 2026 Apr 30;23:63. doi: 10.1186/s12966-026-01927-8 (PMC13277215; doi:10.1186/s12966-026-01927-8)
Supplement: Supplementary file 3 — Additional file 3. [file 12966_2026_1927_MOESM3_ESM.pdf]

Study_ID_number:

Coder_1:
- Kelsay Corlew (1)
- Laura Flournoy (2)
- Emily Shaw (3)
- Shelly Palmer (5)
- Reconciled (4)

Title:

Abstract:

Authors:

Journal:

Publication_year:

Link:

Title_abstract_eligibility:
- Yes (1)
- No (2)

Exclusion_Reason:
- Not an original peer-reviewed article, theses, or dissertation (1)
- Took place outside of the US Cooperative Extension System (2)
- Does not investigate determinants of integrating a PA policy, systems, or environment intervention (3)
- Does not include barriers, facilitators, or contextual factors to integration (4)
- Is not in English (5)
- Published before 2014 (6)
- Is a duplicate (7)
- N/A (0)

Full_eligibility:
- Yes (1)
- No (2)

Full_Exclusion_Reason:
- Not an original peer-reviewed article, theses, or dissertation (1)
- Took place outside of the US Cooperative Extension System (2)
- Does not investigate determinants of integrating a PA policy, systems, or environment intervention (3)
- Does not include barriers, facilitators, or contextual factors to integration (4)
- Is not in English (5)
- Published before 2014 (6)
- Is a duplicate (7)
- N/A (0)

Setting and methods
Funding: How the study was funded
Text if applicable
No funding reported (999)

State: Name of state where the study was conducted
Text if applicable (two letter code)
Not mentioned (999)

Rurality: Area the study was conducted, as reported by authors
Urban (1)
Rural (2)
Not mentioned (999)

Study_Type:
Qualitative (1)
Quantitative (2)
Mixed methods (3)

Intervention
Intervention: type of intervention the study investigating (what were the respondents were asked about)
Physical activity and nutrition PSEs (broad category that includes many different interventions) (1)
Physical activity PSEs or built environment approaches (broad category that includes many different physical activity interventions) (2)
A specific physical activity intervention (3)

Intervention_specific: If "A specific physical activity intervention" was selected, describe the intervention.
Text if applicable
Not described (999)

Program_SNAP-Ed: Were the respondents asked about implementing the intervention as part of SNAP-Ed programming?
Yes (1)
Not specified (2)

Program_EFNEP: Were the respondents asked about implementing the intervention as part of EFNEP programming?
Yes (1)
Not specified (2)

Program_HOP: Were the respondents asked about implementing the intervention as part of HOP (High Obesity Program) programming?
Yes (1)
Not specified (2)

Program_general: Were the respondents asked about implementing the intervention as part of general Extension programming (using general funds; not specifically funded by SNAP-Ed, EFNEP, or HOP funding)?

Yes (1)
Not specified (2)

Determinants

Audience_Community: Were data collected from community members (people who are reached or exposed to the PSE intervention)?

Yes (1)
No (2)

Audience_Agents: Were data collected from Extension Educators or Agents?

Yes (1)
No (2)

Audience_Administrators: Were data collected from administrators?

Yes (1)
No (2)

Audience_Partners: Were data collected from community partners (people who are involved with program implementation)?

Yes (1)
No (2)

Audience_Other: Were data collected from other audiences than community members, Educators/Agents, administrators, or partners?

Yes (1)
No (2)

Audience_Other_Describe:

Text if applicable
Not described (999)

Theory_model_framework: Was a determinants model or framework used?

Yes (1)
No (2)

Theory_Name: If a determinants model or framework was used, what is the name?

PARIHS (Promoting Action on Research Implementation in Health Services) (1)
TDF (Theoretical Domains Framework) (2)
PRISM (Practical, Robust Implementation and Sustainability Model), (3)
CFIR (Consolidated Framework for Implementation Research) (4)
TICD (Tailored Implementation for Chronic Disease) (5)
Other (6)
Atheoretical or not mentioned (999)

Theory_Name_Other

Text if other
Not applicable (999)

Data_Collection_Domains: If a determinants model or framework was used, was data collected on specific domains?

Yes (1)
No (2)
Not applicable (999)

Data_Collection_Individual: Was data collected on the individual level domain?

Yes (1)
No (2)
Not applicable (999)

Data_Collection_Innovation: Was data collected on the innovation level domain?

Yes (1)
No (2)
Not applicable (999)

Data_Collection_Inner: Was data collected on the inner setting level domain?

Yes (1)
No (2)
Not applicable (999)

Data_Collection_Outer: Was data collected on the outer setting/environment level domain?

Yes (1)
No (2)
Not applicable (999)

Data_Collection_Process: Was data collected on the implementation process level domain?

Yes (1)
No (2)
Not applicable (999)

Data_Collection_Other: Was data collected on other domains besides individual, innovation, inner setting, outer setting, or process?

Yes (1)
No (2)
Not applicable (999)

Data_Collection_Other_Describe: Please describe the other domains that data were collected on.

Text if other
Not applicable (999)
Not applicable (999)

Theory_Domain_Individual: Are results for the individual level domain included? Complete this item whether or not a determinants model or framework was used for data collection. (See the CFIR publication for what constructs are included for each level/domain.)

Yes (1)
No (2)

Barriers_Individual: What barriers were identified within the individual level domain? Add a code in parenthesis for the most relevant CFIR construct.

Text if applicable

No barriers identified (999)

Facilitators_Individual: What facilitators were identified within the individual level domain? Add a code in parenthesis for the most relevant CFIR construct.

Text if applicable

No facilitators identified (999)

Theory_Domain_Innovation: Are results for the innovation level domain included? Complete this item whether or not a determinants model or framework was used for data collection.

Yes (1)

No (2)

Barriers_Innovation: What barriers were identified within the innovation level domain? Add a code in parenthesis for the most relevant CFIR construct.

Text if applicable

No barriers identified (999)

Facilitators_Innovation: What facilitators were identified within the innovation level domain? Add a code in parenthesis for the most relevant CFIR construct.

Text if applicable

No facilitators identified (999)

Theory_Domain_Inner: Are results for the inner setting level domain included? Complete this item whether or not a determinants model or framework was used for data collection.

Yes (1)

No (2)

Barriers_Inner: What barriers were identified within the inner setting level domain? Add a code in parenthesis for the most relevant CFIR construct.

Text if applicable

No barriers identified (999)

Facilitators_Inner: What facilitators were identified within the inner setting level domain? Add a code in parenthesis for the most relevant CFIR construct.

Text if applicable

No facilitators identified (999)

Theory_Domain_Outer: Are results for the outer setting/environment level domain included? Complete this item whether or not a determinants model or framework was used for data collection.

Yes (1)

No (2)

Barriers_Outer: What barriers were identified within the outer setting level domain? Add a code in parenthesis for the most relevant CFIR construct.

Text if applicable

No barriers identified (999)

Facilitators_Outer: What facilitators were identified within the outer setting level domain? Add a code in parenthesis for the most relevant CFIR construct.

Text if applicable

No facilitators identified (999)

Theory_Domain_process: Are results for the implementation process level domain included? Complete this item whether or not a determinants theory, model, or framework was used for data collection.

Yes (1)

No (2)

Barriers_Process: What barriers were identified within the implementation process level domain? Add a code in parenthesis for the most relevant CFIR construct.

Text if applicable

No barriers identified (999)

Facilitators_Process: What facilitators were identified within the implementation process level domain? Add a code in parenthesis for the most relevant CFIR construct.

Text if applicable

No facilitators identified (999)

Theory_Domain_Other: Are results for other domains besides individual, innovation, inner setting, outer setting, or process included?

Yes (1)

No (2)

Theory_Domain_Other_Describe: Please describe the other domains that were included in the results.

Text if other

Not applicable (999)

Barriers_Other: What barriers were identified within the other domains?

Text if applicable

No barriers identified (999)

Facilitators_Other: What facilitators were identified within the other domains?

Text if applicable

No facilitators identified (999)

Implementation Strategies

Implementation_Strategies: Were implementation strategies identified?

Yes (1)

No (2)

I_S_Describe: Describe the implementation strategies that were identified (as described by the authors). Add a code in parenthesis of the most relevant implementation strategy from the ISAC compilation.

Text if applicable

Not applicable (999)

I_S_Selection: How were implementation strategies selected (e.g., qualitative study finding, barrier-facilitator matching process, etc.)?

Text if applicable

Not described (999)

Mechanisms: Were the implementation strategies' mechanisms of action (i.e., how they work, such as changing knowledge or confidence) described?

Text if applicable

Not described (999)

Outcomes

Purpose: What is the purpose of the study? Use the aims as stated by the authors.

Text if applicable

Not described (999)

Outcome_Reach: Was investigating how to improve reach a goal of this study? Only include if mentioned verbatim.

Yes (1)

No (2)

Reach: If investigating how to improve reach is a goal, how is it defined?

Text if applicable

Not described (999)

Outcome_Adoption: Was investigating how to improve adoption a goal of this study? Only include if mentioned verbatim.

Yes (1)

No (2)

Adoption: If investigating how to improve adoption is a goal, how is it defined?

Text if applicable

Not described (999)

Outcome_Implementation: Was investigating how to improve implementation a goal of this study? Only include if mentioned verbatim.

Yes (1)

No (2)

Implementation: If investigating how to improve implementation is a goal, how is it defined?

Text if applicable

Not described (999)

Outcome_Maintenance: Was investigating how to improve maintenance or sustainment a goal of this study? Only include if maintenance or sustainment or sustainability are mentioned verbatim.

Yes (1)

No (2)

Maintenance: If investigating how to improve maintenance is a goal, how is it defined?
Text if applicable
Not described (999)

Outcome_Scale: Was investigating how to improve scale-out a goal of this study? Only include if scaling, scale-out, or scale-up are mentioned verbatim.
Yes (1)
No (2)

Scale: If investigating how to improve scaling is a goal, how is it defined?
Text if applicable
Not described (999)

Outcome_Acceptability: Was investigating how to improve acceptability of physical activity PSEs a goal of this study? Only include if mentioned verbatim.
Yes (1)
No (2)

Acceptability: If investigating how to improve acceptability is a goal, how is it defined?
Text if applicable
Not described (999)

Outcome_Appropriateness: Was investigating how to improve appropriateness of physical activity PSEs a goal of this study? Only include if mentioned verbatim.
Yes (1)
No (2)

Appropriateness: If investigating how to improve appropriateness is a goal, how is it defined?
Text if applicable
Not described (999)

Outcome_Cost: Was investigating how to improve cost of physical activity PSEs a goal of this study? Only include if mentioned verbatim.
Yes (1)
No (2)

Cost: If investigating how to improve cost is a goal, how is it defined?
Text if applicable
Not described (999)

Outcome_Feasibility: Was investigating how to improve feasibility of physical activity PSEs a goal of this study? Only include if mentioned verbatim.
Yes (1)
No (2)

Feasibility: If investigating how to improve feasibility is a goal, how is it defined?
Text if applicable
Not described (999)

Outcome_Fidelity: Was investigating how to improve fidelity of physical activity PSEs a goal of this study? Only include if mentioned verbatim.

Yes (1)
No (2)

Fidelity: If investigating how to improve feasibility is a goal, how is it defined?

Text if applicable
Not described (999)

Outcome_Penetration: Was investigating how to improve penetration of physical activity PSEs a goal of this study? Only include if mentioned verbatim.

Yes (1)
No (2)

Penetration: If investigating how to improve penetration is a goal, how is it defined?

Text if applicable
Not described (999)

Outcome_Other_Describe: Please describe other implementation outcomes that the study investigated how to improve.

Text if applicable
Not applicable (999)

MMAT_S1: Are there clear research questions?

Yes
No
Can't Tell

MMAT_S2: Do the collected data allow to address the research questions?

Yes
No
Can't Tell

MMAT_1.1: Is the qualitative approach appropriate to answer the research question?

Yes
No
Can't Tell

MMAT_1.2: Are the qualitative data collection methods adequate to address the research question?

Yes
No
Can't Tell

MMAT_1.3: Are the findings adequately derived from the data?

Yes
No
Can't Tell

MMAT_1.4: Is the interpretation of results sufficiently substantiated by data?

Yes
No
Can't Tell

MMAT_1.5: Is there coherence between qualitative data sources, collection, analysis, and interpretation?

Yes

No
Can’t Tell
MMAT_2.1: Is randomization appropriately performed?
Yes
No
Can’t Tell
MMAT_2.2: Are the groups comparable at baseline?
Yes
No
Can’t Tell
MMAT_2.3: Are there complete outcome data?
Yes
No
Can’t Tell
MMAT_2.4: Are outcome assessors blinded to the intervention provided?
Yes
No
Can’t Tell
MMAT_2.5: Did the participants adhere to the assigned intervention?
Yes
No
Can’t Tell
MMAT_3.1: Are the participants representative of the target population?
Yes
No
Can’t Tell
MMAT_3.2: Are measurements appropriate regarding both the outcome and intervention?
Yes
No
Can’t Tell
MMAT_3.3: Are there complete outcome data?
Yes
No
Can’t Tell
MMAT_3.4: Are the confounders accounted for in the design and analysis?
Yes
No
Can’t Tell
MMAT_3.5: During the study period, is the intervention administered as intended?
Yes
No
Can’t Tell
MMAT_4.1: Is the sampling strategy relevant to address the research question?
Yes
No
Can’t Tell
MMAT_4.2: Is the sample representative of the target population?
Yes
No
Can’t Tell

MMAT_4.3: Are the measurements appropriate?
Yes
No
Can't Tell
MMAT_4.4: Is the risk of nonresponse bias low?
Yes
No
Can't Tell
MMAT_4.5: Is the statistical analysis appropriate to answer the research question?
Yes
No
Can't Tell
MMAT_5.1: Is there an adequate rationale for using a mixed methods design to address the research question?
Yes
No
Can't Tell
MMAT_5.2: Are the different components of the study effectively integrated to answer the research question?
Yes
No
Can't Tell
MMAT_5.3: Are the outputs of the integration of qualitative and quantitative components adequately interpreted?
Yes
No
Can't Tell
MMAT_5.4: Are divergences and inconsistencies between quantitative and qualitative results adequately addressed?
Yes
No
Can't Tell
MMAT_5.5: Do the different components of the study adhere to the quality criteria of each tradition of the methods involved?
Yes
No
Can't Tell
